# Exploring the Effects of Immersive Virtual Reality Environments on Sensory Perception of Beef Steaks and Chocolate

**DOI:** 10.3390/foods10061154

**Published:** 2021-05-21

**Authors:** Emily Crofton, Niall Murray, Cristina Botinestean

**Affiliations:** 1 Teagasc Food Research Centre, Department of Food Quality and Sensory Science, D15 KN3K Dublin, Ireland; cristina.botinestean@teagasc.ie; 2 Athlone Institute of Technology, Faculty of Engineering and Informatics, Department of Computer and Software Engineering, N37 F6D7 Athlone, Ireland; nmurray@research.ait.ie

**Keywords:** beef, chocolate, acceptability, virtual reality, immersive environments

## Abstract

Virtual reality (VR) technology is emerging as a tool for simulating different eating environments to better understand consumer sensory response to food. This research explored the impact of different environmental contexts on participants’ hedonic ratings of two different food products: beef steaks, and milk chocolate, using VR as the context-enhancing technology. Two separate studies were conducted. For beef, two different contextual conditions were compared: traditional sensory booths and a VR restaurant. For chocolate, data were generated under three different contextual conditions: traditional sensory booths, VR Irish countryside; VR busy city (Dublin, Ireland). All VR experiences were 360-degree video based. Consumer level of engagement in the different contextual settings was also investigated. The results showed that VR had a significant effect on participants’ hedonic responses to the food products. Beef was rated significantly higher in terms of liking for all sensory attributes when consumed in the VR restaurant. While for chocolate, the VR countryside context generated significantly higher hedonic scores for flavour and overall liking in comparison to the sensory booth. Taken together, both studies demonstrate how specific contextual settings can impact participants’ sensory response to food products, when compared to a traditional sensory laboratory condition.

## 1. Introduction

The act of eating is among the most pleasurable and enjoyable experiences in our everyday lives. It is also a highly multisensory experience involving a dynamic interplay between the brain and each of the five senses, comprising visual and auditory cues, smells, tastes, and tactile sensations. While much of the pleasure we perceive from food depends on the quality of the ingredients and how they are prepared, our surrounding environment or context in which we consume the food also plays a critical role. The location or setting, the type of music, lighting or sounds, and the presence or not of family and friends, are all examples of contextual factors that consumers typically consider when making sensory judgements about a food or a meal [1,2,3].

Despite consumption context playing an important role in how people perceive food, consumer-based sensory tests typically take place in a highly controlled sensory laboratory setting, which lacks environmental or contextual cues. While a sensory testing facility is specifically designed to minimise potential variation caused by the external environment, the setting does not represent how consumers interact with food products in real life [4]. In addition, and beyond contextual cues, a sterile sensory laboratory setting may also induce feelings of boredom, disinterest, and a lack of engagement among participants, resulting in data that poorly predicts the success rate of newly launched products in the marketplace. Although many factors can contribute to the poor predictive validity of consumer sensory data, researchers have cited an overall lack of effective engagement by study participants during the sensory task as a contributing cause [2,5,6]. As a result, there is considerable interest among researchers in investigating ways to evoke consumption contexts during the sensory evaluation of foods, with the aim of improving the ecological validity of consumer data. To date, several different approaches have been proposed including written, imagined or image depicted scenarios [4,7], the creation of an immersive environment, such as a beach [8] or café [9], and the use of virtual reality technology [10,11,12].

The use of virtual reality (VR) technology to simulate immersive contextual settings for hedonic testing is becoming an increasingly popular study area in sensory science (see Crofton et al. [13] for a review describing recent advancements in virtual and augmented reality and their potential application in sensory science). Research comparing consumer responses generated in immersive VR environments and sensory laboratory settings have been recently reported for a range of food products, including chocolate [11], tea break snacks [14], cheese [15], and alcoholic beverages [10,12]. Consumer hedonic ratings were found to change depending on the context for some products [12,15], but not others [10,11], although this may be explained by the different environmental contexts used across these studies. Outside of academia, the food industry are exploring ways VR technology can be used to customise a unique multisensory experience for their consumers. For instance, Guinness, a popular Irish stout sold worldwide, recently designed specific VR environments to complement the sensory profile of a new range of beers [16], while restaurants are adopting the technology to create more memorable and engaging food experiences [17].

The potential for VR technology to generate ecologically valid data comparable to that obtained in a natural consumption setting, in a faster, more cost-effective and controlled experimental manner, is a promising innovation strategy for the food industry to consider [14,18]. In this regard, the overall aim of this research was to explore the effect of different environmental contexts on consumers’ hedonic responses to two different food products: beef, and milk chocolate, using VR as the context-enhancing technology. Beef and chocolate are associated with very different types of eating occasions. For instance, beef is typically eaten in the context of a meal, while chocolate is considered a snack suited to a range of eating occasions [11,19]. As certain products are better suited to specific situations of consumption [20], our hypothesis was that a virtual environment appropriate to each product, may enhance consumer hedonic ratings, when compared to sensory testing booths as the control setting. Therefore, two separate studies were conducted. For beef, two different contextual conditions were compared; (1) traditional sensory booth; (2) VR restaurant. For chocolate, data were generated under three different contextual conditions; (1) traditional sensory booth; (2) VR Irish countryside; (3) VR busy city (Dublin, Ireland). Consumer level of engagement in the different contextual settings was also investigated.

## 2. Materials and Methods

### 2.1. Participants

Participants for both the beef trial (*n* = 30, 15 females and 15 males, ranging in age from 22 to 55 years) and the chocolate trial (*n* = 30, 18 females and 12 males, ranging in age from 22 to 65 years) were recruited via internal email from a pool of staff and students based at Teagasc Food Research Centre, Dublin, Ireland. Participants had to meet the following inclusion criteria: (1) consumed the product used in the study at least twice per month; (2) had no known history of food allergies related to the study products; and (3) did not suffer from motion sickness. Selected participants were asked to refrain from eating, drinking, or smoking for at least 1 h prior to the start of the trial. Informed consent was obtained from each participant and they were free to withdraw from the study at any time. The sensory trials were conducted in accordance with the guidelines for ethical and professional practices for the sensory analysis of foods as set out by the Institute of Food Science and Technology [21] and the American Meat Science Association [22]. The sessions were conducted during mid-morning (10:00–11:30 a.m.) and took place at the Sensory Science Suite at Teagasc Food Research Centre, which has been designed in accordance with ISO 8586:2007.

### 2.2. Product Stimuli and Sensory Procedure

#### 2.2.1. Beef Trial

Individual beef steaks of 2.54 cm thickness were cut from the M. longissimus lumborum. Each beef steak was vacuum-packaged and stored at −20 °C until analysis. The steaks were placed in a refrigerator to thaw 24 h prior to sensory analysis. One hour prior to cooking, the steaks were taken from the refrigerator and removed from the vacuum bag. Steaks were cooked on a Velox grill using minimal cooking oil and no seasoning, until an internal temperature of 71 °C was reached [22]. Each steak was removed from the grill, allowed to rest for 2 min, wrapped in aluminium foil and labelled with a random three-digit code. Each steak was cut into two equal-sized portions and presented monadically to participants in two different environments: a traditional sensory booth and a virtual reality restaurant. The presentation order of the environmental context was completely balanced across participants (i.e., 15 participants assessed the beef in the traditional booth first, and 15 in the virtual reality restaurant first). Participants were instructed to evaluate the meat for liking in terms of smell, tenderness, juiciness, beef flavour, and overall liking on a 9-point hedonic scale, where 1 = dislike extremely and 9 = like extremely. In an effort to retain the level of immersion in the virtual reality condition, participants provided answers to the questions verbally in all conditions, and the data was immediately recorded by the researchers via Compusense Cloud^®^ (West Guelph, ON, Canada). All participants were familiarised with the questionnaire design and testing procedure prior to starting the trial and were instructed on how to wear and use the VR headsets. Beef samples were served to participants after they were wearing the HMD device, and researchers were available to assist the participants, if required. A five-minute break was enforced between each tasting condition and plain crackers (Carr’s, UK) and mineral water (Evian, France) were provided as palate cleansers to avoid product carry-over effects.

#### 2.2.2. Chocolate Trial

Milk chocolate (20% cocoa, Cadbury’s) was the sensory stimuli used in this trial. It was purchased at a local convenience store and was stored at room temperature prior to the sensory trial. Three identical square pieces of chocolate, taken from the same chocolate bar, were placed on separate ceramic white plates 5 min prior to each testing session, and presented monadically to participants in three testing environments—a traditional sensory booth, a VR Irish countryside, and a VR busy city. The presentation order of the testing environment was completely balanced across participants. Participants were instructed to evaluate the chocolate for liking in terms of smell, flavour, sweetness, texture, smoothness, and overall liking on a 9-point hedonic scale, where 1 = dislike extremely and 9 = like extremely. Similar to the approach taken in the beef trial, participants provided answers to the questions verbally in all conditions in an effort to retain the degree of perceived immersion in the VR session. All participants were familiarised with testing procedures prior to starting the test. Data was recorded by the researchers via Compusense Cloud ^®^ (West Guelph, ON, Canada). Plain crackers (Carr’s, UK) and mineral water (Evian, France) were used as palate cleansers and a five-minute break was enforced between each tasting condition to avoid product carry-over effects.

### 2.3. Contextual Settings

#### 2.3.1. Beef Trial

**Figure 1:**
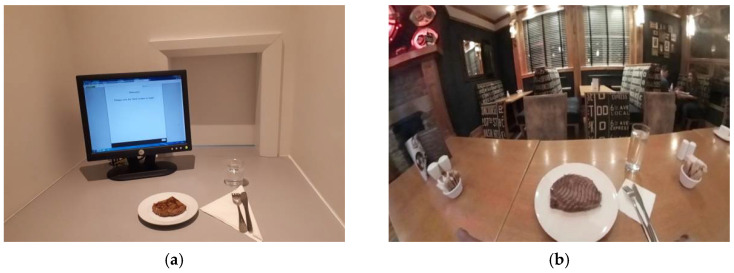
Contextual settings for the sensory evaluation of beef samples: (**a**) Traditional sensory booth; (**b**) Virtual Reality (VR) restaurant.

Two different experimental conditions were set-up; a traditional sensory booth (Figure 1a) and an immersive VR restaurant (Figure 1b). The VR restaurant context was captured using a Samsung Gear 360 4 K Ultra High Definition (HD) camera at a restaurant in Dublin, Ireland. The restaurant was open to customers during the recording to ensure the atmosphere created was as close as possible to real-life conditions. Audio recordings consisting of indistinguishable conversation and background noises were also recorded. The 360-degree video was 2 min in length and was set to loop throughout the tasting condition. The video was presented to participants through a head mounted display (HMD) (Oculus Go).

#### 2.3.2. Chocolate Trial

**Figure 2:**
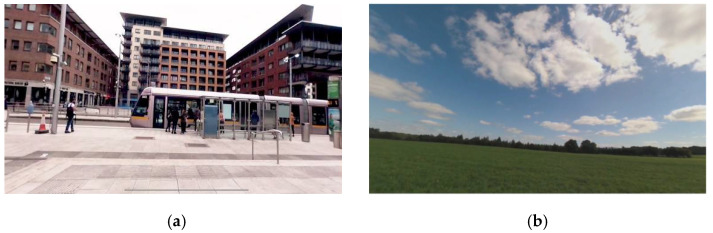
Contextual settings for the sensory evaluation of chocolate: (**a**) VR busy city (Dublin, Ireland) and (**b**) VR Irish countryside.

Participants evaluated chocolate in three different environmental contexts: a traditional sensory booth, a VR busy city (Figure 2a), and a VR Irish countryside (Figure 2b). These specific VR contexts were chosen as they were expected to elicit different hedonic responses from participants. The VR environments were created using 360-degree videos displayed through a HMD (Oculus Go). The VR videos were captured using a Garmin VIRB 360 camera and included audio recordings. Both 360-degree videos had a length of 2 min and the videos were set to loop during each tasting session. The 360-degree busy city video was recorded in Dublin City, Ireland, and depicted the International Financial Services Centre (IFSC), public transport, and people walking through the streets. The video was supported by the sounds of the busy city environment. The 360-degree countryside video was recorded in a rural part of Meath, Ireland and provided a view of a typical Irish countryside setting with bright sunlight, the movement of the grass and the background noises included the sound of the wind.

For both trials, participants were asked questions regarding their level of engagement, perceived effort to assess the samples, level of distraction, and purchase intent. The following questions were asked for all contextual environments: Was the sensory testing experience memorable? Do you think testing the product in the surrounding environment requires much effort? Do you think the surrounding environment distracted you from performing the task? Responses to these questions were rated on a 7-point scale where 1 = not at all and 7 = very much. Purchase intent of each sample was measured using 5-point scale where 1 = definitely not buy and 5 = definitely buy.

### 2.4. Data Analysis

Due to the small sample size of participants used in both the beef (*n* = 30) and chocolate (*n* = 30) trial, and because the data collected was ordinal, non-parametric tests were used to analyse the data. For the chocolate trial, the Friedman’s ANOVA test was performed to determine the effect of the different environmental conditions on participants’ hedonic ratings of chocolate samples. Where Friedman’s indicated significant differences were present, a Wilcoxon pairwise comparison (with Bonferroni adjustment) was conducted to identify where the differences occurred. In terms of the beef trial, a Wilcoxon signed-rank test was conducted to test if the surrounding environment influenced hedonic ratings of beef samples. The probability level used for significance between responses was set at *p* < 0.05 and all data were analysed using SPSS 25 statistical software (IBM, Armonk, NY, USA).

## 3. Results and Discussion

### 3.1. The Effect of the Surrounding Environment on Participants’ Hedonic Ratings of Beef Steaks

Tenderness, juiciness, and flavour are the most important palatability attributes affecting beef eating quality [23,24]. In this trial, participants evaluated beef steak in terms of smell, tenderness, juiciness, flavour, and overall liking in two different contextual settings. Participants were unaware that the sample of beef presented in both settings was taken from the same steak. A Wilcoxon signed-rank test showed that the environmental condition had a significant effect of liking of attributes smell (Z = −2.514, *p* = 0.012), tenderness (Z = −3.094, *p* = 0.002), juiciness (Z = −3.129, *p* = 0.002) and flavour (Z = −2.982, *p* = 0.003), with beef consumed in the VR restaurant environment setting receiving higher liking scores, compared to the sensory booths. In addition, overall liking scores were significantly greater (Z = −2.898, *p* = 0.004) for beef eaten in the VR context, indicating that the VR restaurant had a positive impact on participants’ hedonic ratings of beef steaks. Descriptive statistics for the data is presented in Table 1. To the best of our knowledge, no studies to date have investigated the application of immersive technologies, such as VR, in meat sensory research. Hersleth et al., (2015) [25], showed that evoked meal contexts in the form of written texts and images, affected consumers’ responses to both the intrinsic and extrinsic attributes in dry-cured ham, although the strongest effects were observed for the extrinsic ratings. In addition, many studies have demonstrated that a restaurant environment is typically associated with positive emotions [26,27,28], and that consumers tend to be more critical of beef tenderness in the home than in a restaurant [24]. Taken together, these findings indicate that the surrounding environment can considerably influence consumers’ sensory perception of beef. As a result, VR technology could provide the food industry with new opportunities to improve sensory marketing efforts through the creation of immersive, multisensory dining experiences, specifically designed to enhance consumer engagement.

The potential for VR in creating a unique eating experience has never been more relevant, as the current coronavirus pandemic continues to hamper access to sharing meal experiences with family and friends. In addition, recent advancements in virtual reality and computational gastronomy could be used an as innovative tool to create multisensory meat-eating experiences while potentially alleviating the health concerns consumers often associate with red meat [13,29]. A more realistic insight into the environmental or contextual cues which influence sensory perception of meat, may also assist industry in developing innovative strategies that will assist in improving the meat-eating experience, and enhance brand characteristics by eliciting stronger emotional connections towards meat products.

### 3.2. The Effect of the Surrounding Environment on Participants’ Hedonic Ratings of Chocolate Samples

In this study, participants rated the smell, flavour, sweetness, texture, smoothness, and overall liking of three identical pieces of chocolate in three different contextual settings. Participants were unaware that the same chocolate was being served in all three conditions. There was a significant difference in perceived liking of flavour (χ^2^(2) = 7.971, *p* = 0.019) and overall liking (χ^2^(2) = 10.750, *p* = 0.005), depending on the environment in which the chocolate was consumed. A post hoc analysis with Wilcoxon signed-rank tests was conducted with a Bonferroni correction applied, resulting in a significance level set at *p* < 0.017. The results showed that participants rated the flavour (Z = −2.950, *p* = 0.003) and overall liking (Z = −2.885, *p* = 0.004) of chocolate significantly higher when consumed in the VR countryside setting, compared to the traditional sensory booth. The surrounding context did not influence hedonic ratings of other chocolate attributes. However, in general, the VR countryside setting produced higher liking scores for chocolate compared to the VR busy city and sensory booth environment. Descriptive statistics for the data is presented in Table 2.

While our research findings agree with previous studies which show that consumers’ hedonic ratings of food can change depending on the specific context [2,12,15,25,26], it is also contradictory with others [8,10,11]. For instance, Kong et al. (2020) [11] studied the impact of three contextual settings, including sensory booths and two VR environments (a pleasant sightseeing tour and a live music concert), on consumer liking of three types of chocolate. While their research showed significant differences in consumer liking of the different chocolate types, the surrounding context did not influence the tasting experience. The different immersive environments used to evoke context might explain the inconsistency in findings between this study and ours. Research suggests that certain food products are better suited to specific consumption contexts [3,30], which in turn may alter the sensory perception of food during consumption, through the elicitation of a positive emotional response [26]. Although in this exploratory study we did not measure consumer emotional response to chocolate in the different immersive environments, the more naturalistic and peaceful setting of the Irish countryside might have improved the mood of the participants, which favourably influenced their liking of the chocolate flavour, when compared to the sensory booths and VR city environment [31]. Based on this assumption, we would have expected the chocolate consumed in the busy VR city environment to have produced overall lower hedonic ratings in comparison to the quiet sensory booth setting, as observed elsewhere for chocolate ice-cream [26]. Picket and Dando [12], used VR to investigate how the surrounding environment (winery vs bar) influenced sensory perception of two alcoholic drinks (wine and beer). They showed that while participants liked the wine more and were willing to pay more for it when it was consumed in a virtual winery context, these effects were not observed for beer. 

VR technology is a promising tool for simulating immersive environments for consumer sensory evaluations and could be used in conjunction with non-invasive biometric sensors to enrich our understanding of the emotions responsible for driving consumer liking in the real world [2,13,32]. Nonetheless, as research in this area is in its infancy, further studies are required to identify the specific conditions in which utilizing VR is relevant and is likely to improve the reliability and ecological validity of sensory testing outcomes [2,13].

Beyond the importance of selecting an appropriate immersive environment for a particular food product, another possible explanation for the conflicting results associated with using VR as a context-enhancing technology for sensory assessments could be related to how it is being applied during sensory tests. For instance, some studies, including ours, have created immersive VR environments by displaying custom-recorded 360-degree VR videos and their corresponding sounds through a HMD [11,12,15]. In contrast, computer-simulated 3D virtual environments, in which participants can physically walk around and interact with the virtual space using a HMD, have been used to study various aspects of consumer behaviour [33,34]. While both methods are expected to provide a more engaging experience compared to a traditional testing environment [2,5], the use of the HMD fully replaces the participants’ view of the real world, restricting their ability to interact visually with the ‘real’ food product. In an effort to capture consumers’ hedonic responses while fully immersed in the VR environment, the participants in this study provided answers to the questions verbally, which were subsequently recorded by the researcher. In similar studies, participants were instructed to remove their VR headsets after tasting and answer questions on a paper ballot [10,11], while othersoverlaid audio instructions and visual scales in the 360-degree VR video [15]. While each approach has clear advantages and disadvantages, future studies could investigate which approach can best improve the ecological validity of consumer sensory data, while continuing to retain participant engagement. In the meantime, this limitation could be overcome by using a mixed-reality device, such as Microsoft HoloLens, which integrates specific elements from the ‘real-world’ within the surrounding VR space, enabling the user to interact with the real food product while simultaneously answering questions in a controlled environment [13]. Mixed reality was recently utilised to understand consumer response to tea-break snacks and was shown to evoke ecologically valid data comparable to a real-life context [14].

### 3.3. Engagement, Effort, Distraction and Purchase Intent for Beef Steaks and Chocolate

**Figure 3:**
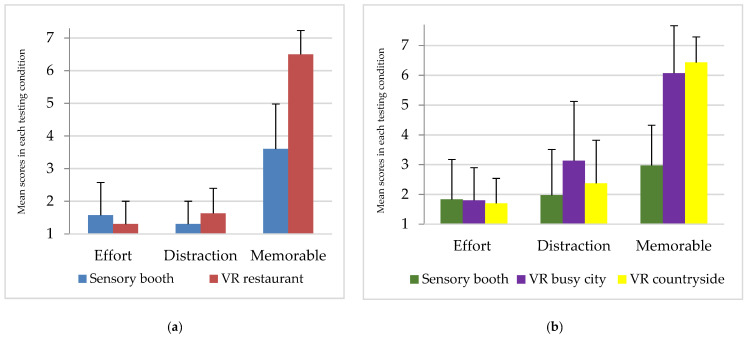
Participants mean scores and standard deviations for beef (**a**) and chocolate (**b**) in each testing condition. The questions asked were: Do you think testing the product in the surrounding environment requires much effort? Do you think the surrounding environment distracted you from performing the task? Was the sensory testing experience memorable? Responses recorded on a 7-point scale where 1 = not at all and 7 = very much.

The mean scores and standard deviations for beef and chocolate are shown in Figure 3a,b, respectively. There were no significant differences in the effort required to perform the sensory test in the respective environments for beef (*p* = 0.865) or chocolate (*p* = 0.153). For beef, a significant difference (Z = −2.262, *p* = 0.013) was found in terms of distraction between the two environments, with participants recording higher levels of distraction when immersed in the VR restaurant setting than the sensory booths. This effect was not observed in the chocolate study, although the effect was approaching significance (*p* = 0.055), with scores indicating that the VR busy city environment was more distracting than the sensory booth. Two separate studies by the same research group found contrasting results with respect to the impact of immersion on levels of distraction [2,5]. However, in these studies, the immersive context was presented to participants by means of a purpose-built physical environment, as opposed to a VR headset as used in ours. Nonetheless, efforts to minimise potential distractions contained within an immersive VR environment is required to improve the value and reliability of data generated using this method.

In our study, participants found the VR restaurant experience to be a significantly more memorable testing environment in comparison to evaluating the beef steaks in the traditional sensory booth set-up (Z = −4.323, *p* < 0.0001). A similar trend was observed for the chocolate samples tested in the three different environments, with the VR settings perceived as more memorable compared to the traditional sensory booth (χ^2^(2) = 46.444, *p* < 0.0001). In terms of purchase intent, no significant differences were observed between the three environmental conditions for chocolate (*p* = 0.124). For beef, participants were more willing to purchase the beef when consumed in the VR restaurant than in the sensory booth (*p* < 0.05). 

### 3.4. Limitations

While our data yielded some interesting findings with respect to how different VR environments impact hedonic response to beef and chocolate, our study has some limitations that offer avenues for further research. An obvious limitation is the relatively small sample size of participants used in both experimental trials, and therefore further research is needed in a more representative sample of the population. Another potential limitation was that participant hedonic responses were not also collected in a ‘real-world’ environment, which was not possible in this research study due to economic and time constraints. Finally, it must be noted that improvements are needed in how data are collected from participants immersed in a VR environment, such as including scales in the VR video, or utilizing a mixed-reality approach, in which elements of the ‘real-world’ environment are integrated within the VR space.

## 4. Conclusions

This study demonstrated that changing the surrounding environment had a significant effect on participants’ hedonic ratings of beef and chocolate. Specifically, we showed that an immersive VR environment tended to induce a positive hedonic response, when compared to a traditional sensory booth setting. Beef was rated as significantly higher for all sensory attributes when consumed in the VR restaurant. For chocolate, the VR countryside context generated significantly higher hedonic scores for flavour and overall liking in comparison to the sensory booth. In general, the VR countryside setting produced the highest liking scores for chocolate. This study also demonstrated that the perceived effort to assess food samples was not significantly impacted by using VR technology, although the surrounding VR environment was more distracting, in comparison to the sensory booths. Overall, this research enhances our understanding of how different environments, simulated using VR technology, can impact sensory perception of beef and chocolate. The study also demonstrates how VR technology can be used to evoke contextual information in consumer sensory tests, which is often an expensive and time-consuming task to conduct in a ‘real-world’ environment. Nonetheless, as studies investigating the application of VR in stimulating the human senses are only beginning to emerge, further research is necessary before the technology can be confidently used to improve the predictive validity of consumer sensory assessments.

## Figures and Tables

**Table 1:** Means, standard deviations (SD), medians and interquartile ranges (IQR) for participant hedonic ratings * of beef in a traditional sensory booth and a virtual reality (VR) restaurant environment.

Context/Attribute	Sensory Booth				VR Restaurant			
	Mean	SD	Median	IQR	Mean	SD	Median	IQR
Smell	6.67	1.12	6.5	1.25	7.20	0.85	7	1
Tenderness	6.17	2.02	7	3	7.53	1.48	7	2
Juiciness	5.87	1.85	6	2.25	7.03	1.38	7	2
Flavour	5.93	1.62	6	2	6.87	1.41	7	2
Overall liking	6.13	1.85	7	2.25	7.17	1.12	7	1.25

* Evaluated on a 9-point hedonic scale, where 1 = dislike extremely and 9 = like extremely.

**Table 2:** Means, standard deviations (SD), medians and interquartile ranges (IQR) for participant hedonic rating * of chocolate in a traditional sensory booth, a VR busy city and a VR Irish countryside environment.

Context/Attribute	Sensory Booth				VR Busy City				VR Irish Countryside			
	Mean	SD	Median	IQR	Mean	SD	Median	IQR	Mean	SD	Median	IQR
Smell	6.53	1.78	7	3	6.77	1.46	7	2	7.10	1.35	7	2
Flavour	6.83	1.62	7	2	7.13	1.41	7	2	7.33	1.24	7.5	1.25
Sweetness	7.37	1.22	7	1.5	7.23	1.41	7	1.25	7.13	1.43	7	2.25
Texture	7.03	1.65	7	2.25	6.97	1.25	7	1	7.27	1.23	7	2
Smoothness	7.10	2.04	8	3	7.27	0.98	7	1.25	7.43	1.17	8	1
Overall liking	6.80	1.58	7	2	7.23	1.33	7	2	7.50	1.11	7	1

* Evaluated on a 9-point hedonic scale, where 1 = dislike extremely and 9 = like extremely.

## Data Availability

The data presented in this study is available on request from the corresponding author.
